# Harnessing stress-tolerant plant growth-promoting rhizobacteria for climate-resilient rice agroecosystems

**DOI:** 10.3389/fsysb.2026.1998786

**Published:** 2026-10-27

**Authors:** Ma. del Carmen Orozco-Mosqueda, Victor J. Carrión, Olubukola Oluranti Babalola, Mauricio Schoebitz, Gustavo Santoyo

**Affiliations:** 1 Department of Biochemical and Environmental Engineering, National Technological Institute of Mexico in Celaya, Gto, Celaya, Mexico; 2 Departamento de Microbiología, Facultad de Ciencias, Universidad de Málaga, Málaga, Spain; 3 Área de Protección de Cultivos, Instituto de Hortofruticultura Subtropical y Mediterránea “La Mayora”, Universidad de Málaga-Consejo Superior de Investigaciones Científicas (IHSM-UMA-CSIC), Málaga, Spain; 4 Institute of Biology, Leiden University, Leiden, Netherlands; 5 Department of Microbial Ecology, Netherlands Institute of Ecology (NIOO-KNAW), Wageningen, Netherlands; 6 Soil-Plant-Microbe Interactions Group, Faculty of Natural and Agricultural Science, North-West University, Mmabatho, South Africa; 7 Departamento de Suelos y Recursos Naturales, Facultad de Agronomía, Casilla 160-C, Universidad de Concepción, Concepción, Chile; 8 Centro de Biotecnología, Universidad de Concepción, Barrio Universitario s/n, Concepción, Chile; 9 Institute of Chemical-Biological Research, Universidad Michoacana de San Nicolás de Hidalgo, Morelia, Mexico

**Keywords:** ACC deaminase, *Bacillus*, drought, multiomics approaches, plant microbiome, rice, salinity

## Abstract

Environmental stresses pose a serious threat to global rice production. Drought, flooding, salinity, heavy metal contamination, and the excessive use of synthetic pesticides are among the major stressors affecting rice production, with some of these pressures being exacerbated by climate change. This review examines the ecological roles, mechanisms, and potential applications of stress-tolerant plant growth-promoting rhizobacteria (PGPR) as a sustainable strategy to enhance the resilience of rice agroecosystems under adverse environmental conditions. Particular emphasis is placed on the ecological responses of the rice rhizosphere microbiome and the molecular and physiological responses of rice plants to abiotic stress, together with the adaptive mechanisms associated with stress-tolerant PGPR activity. These include modulation of stress-responsive genes, enhanced antioxidant responses, osmoprotectant production, exopolysaccharide synthesis, and biofilm formation. The role of bacterial ACC deaminase in modulating stress-induced ethylene accumulation in rice is also reviewed. In addition, the potential contribution of stress-tolerant PGPR to mitigating methane emissions from paddy fields is explored, together with the potential of saline and heavy metal-contaminated soils as reservoirs of novel multi-stress-tolerant PGPR. Finally, recent advances in the bioformulation and delivery of stress-tolerant PGPR are discussed, highlighting their potential to improve bacterial survival, rhizosphere colonization, and field performance and to support more sustainable and climate-resilient rice production.

## 1 Introduction

Agricultural production in the 21st century faces various environmental challenges, including climate change and its consequences, such as rising temperatures, extreme weather events, and shifts in rainfall patterns that alter agricultural cycles and affect crops (Habib-ur-Rahman et al., 2022). Alarming data have been published for decades, yet little has been done to mitigate these effects effectively. For instance, since 1750, the concentration of greenhouse gases has increased significantly, with approximate rises of 150% for methane (CH_4_), 40% for carbon dioxide (CO_2_), and 20% for nitrous oxide (N_2_O) (Filonchyk et al., 2024). This suggests that in the next 50–70 years, the average global temperature could rise by 2 °C, which would have a profound impact not only on agriculture but also on human health and the global economy (Kontgis et al., 2019).

Climate change-associated abiotic stressors are adversely affecting rice cultivation, a crop that, along with maize and wheat, forms the foundation of the global food supply. *Oryza sativa* L., in particular, is a vital staple cereal that sustains a large and growing proportion of the world’s population (Kontgis et al., 2019; Wassmann et al., 2009). Rice is a dietary cornerstone for more than half of the world’s population, with annual global production exceeding 750 million tons. Asia dominates its cultivation, with China, India, Indonesia, and Vietnam as the leading producers and exporters. In addition to its role in food security, rice supports the livelihoods of millions of smallholder farmers (Danish et al., 2024). In Latin America, countries such as Brazil, Colombia and Peru, have significant rice production, while in Mexico, rice is mainly grown in localized regions. However, its consumption is widespread and valued for its nutritional content (Cruz et al., 2021; Mohidem et al., 2022; Portalanza et al., 2022).

Unfortunately, rice is highly sensitive to changes in water availability, soil salinity, and extreme temperatures. Furthermore, its cultivation in flooded soils promotes methane emissions, contributing to climate change and creating a vicious cycle of environmental feedback. Therefore, enhancing rice resilience to abiotic stress and adopting sustainable agricultural practices that reduce or eliminate synthetic fertilizers (which also promote nitrous oxide release) and pesticides are urgent priorities (Dar et al., 2021; Sarma et al., 2023).

One of the most effective strategies to achieve dual benefits in crops such as rice is the application of plant growth-promoting rhizobacteria (PGPR), which act as both biofertilizers and biocontrol agents against plant pathogens. Their application has been widely reported as effective, with consistent results observed in laboratory, greenhouse, and open-field conditions. Additionally, PGPR exhibit mechanisms that confer tolerance to abiotic stresses, which commonly limit rice growth and productivity. These bacteria are referred to as stress-tolerant PGPR (Reshma and Dileep, 2025).

Stress-tolerant PGPR represent a key agrobiotechnological tool for mitigating the negative impacts of abiotic stress. These bacteria not only enhance plant nutrition and growth but also improve tolerance to adverse conditions such as salinity, drought, heavy metal toxicity, and heat stress, to name but a few (Mahmud et al., 2023). Some of the most studied genera include *Pseudomonas*, *Bacillus*, *Burkholderia*, *Gluconacetobacter*, *Azospirillum*, *Enterobacter*, and *Serratia* (Mmotla et al., 2025). These genera belong to different taxonomic groups, for example, genus *Pseudomonas* is classified within the phylum *Pseudomonadota*, class *Gammaproteobacteria*, order *Pseudomonadales*, and family Pseudomonadaceae, whereas genus *Bacillus* belongs to the phylum *Bacillota.* These beneficial bacteria can trigger physiological and biochemical responses in plants, such as the production of antioxidant enzymes (e.g., catalase, peroxidase, and superoxide dismutase), accumulation of osmoprotectants (such as proline, trehalose, glycine betaine), and activation of defense-related genes, including those associated with abscisic acid (ABA), a key phytohormone in plant response to drought and salinity (Mishra et al., 2017; Munir et al., 2022). A key mechanism for alleviating abiotic stress (as well as biotic stress such as pathogen attack) is the production of the enzyme 1-aminocyclopropane-1-carboxylic acid (ACC) deaminase. This enzyme lowers ethylene levels in plants by breaking down its precursor, ACC. Since ethylene concentrations rise under abiotic stress and can inhibit plant growth, ACC deaminase activity helps mitigate these negative effects, promoting healthier development (Orozco-Mosqueda et al., 2020). PGPR also function through multiple and complementary mechanisms, including phytohormone production, nutrient solubilization, and synthesis of beneficial volatile compounds (Nabati et al., 2025). Therefore, this review aims to analyze the potential of PGPR, particularly stress-tolerant strains, to mitigate environmental stresses in rice and promote sustainable and climate-resilient rice production.

## 2 Molecular and physiological responses of plants to abiotic stress

Abiotic stress has serious consequences for agriculture, affecting crop yields and farmers’ economic gains. Various crops experience reduced photosynthetic capacity and impaired nutrient acquisition (Figure 1). One of the first plant organs to encounter abiotic stress is the root system, which, under moderate stress conditions, may alter its architecture and biomass to compensate for nutrient or water limitations (Bechtold and Field, 2018; Fromm, 2019). Under more severe stress, rice plants may experience complete growth inhibition, with development coming to a halt. Molecular signaling pathways.involving *STOP1* (a transcription factor), *ALMT1* (a malate transporter channel), and *LPR2* (*LOW PHOSPHATE ROOT 2*) are responsible for arresting root cell elongation (Balzergue et al., 2017). These genes, involved in responses to aluminum stress and phosphate deficiency, have been described in *Arabidopsis thaliana* (L.) Heynh. and *O. sativa* L. (Chen et al., 2022).

**FIGURE 1:**
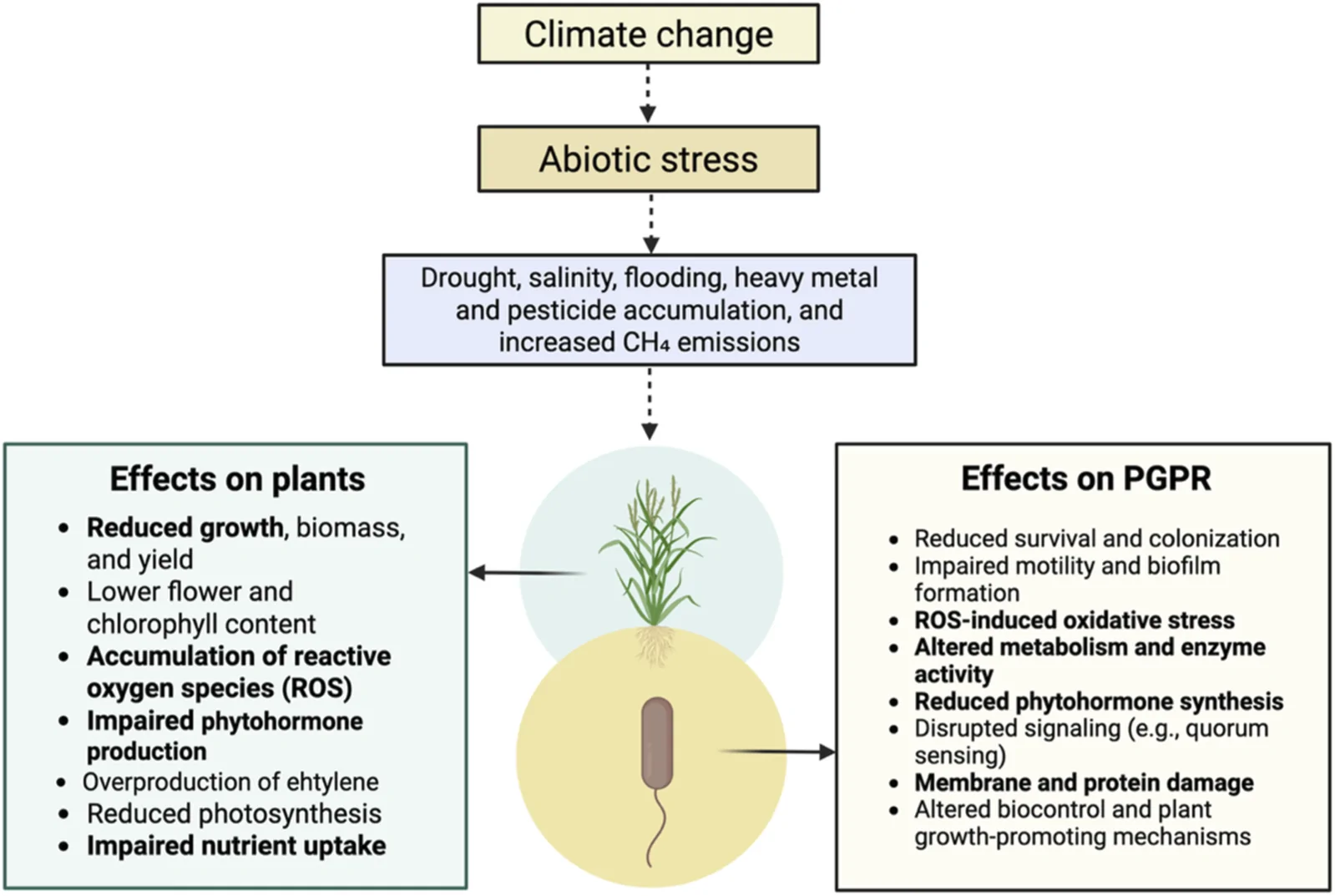
Effect of climate change-induced abiotic factors on plants and plant growth-promoting rhizobacteria. Modified from [19].

Phytohormones are key regulators of plant growth and development and play a central role in plant adaptation to abiotic stress. Under adverse conditions, plants modulate the synthesis and signaling of hormones such as auxins, cytokinins, gibberellins, and brassinosteroids (BR). Auxins, such as indole-3-acetic acid (IAA), promote the formation of lateral roots and root hairs, enhancing water and nutrient uptake, particularly in saline or nutrient-poor soils. Cytokinins stimulate cell division and maintain meristematic tissues while delaying senescence, which is essential to sustain growth under stress. Gibberellins support cell elongation, seed germination, and reproductive development-processes often suppressed under unfavorable conditions (Orozco-Mosqueda et al., 2023; Yin et al., 2025).

A widespread plant response to abiotic stress-whether due to drought, salinity, high temperatures, or heavy metals-is the overproduction of reactive oxygen species (ROS), such as singlet oxygen (^1^O_2_), superoxide (O_2_•^-^), hydrogen peroxide (H_2_O_2_), and hydroxyl radicals (•OH) (Choudhury et al., 2017). Therefore, the regulation of antioxidant defenses is critical for plant survival and includes non-enzymatic mechanisms, such as carotenoids, ascorbate (AsA), glutathione (GSH), tocopherols, and melatonin, which also functions as a hormonal regulator. Enzymatic antioxidant responses include superoxide dismutase (SOD), catalase (CAT), ascorbate peroxidase (APX), dehydroascorbate reductase (DHAR), monodehydroascorbate reductase (MDHAR), glutathione reductase (GR), glutathione S-transferase (GST), glutathione peroxidase (GPX), and other peroxidases (POX) (Fujita and Hasanuzzaman, 2022). Brassinosteroids also play an important role by modulating hormonal balance and enhancing antioxidant responses (Yin et al., 2025).

During drought, plants employ directional root growth mechanisms to move toward areas of higher soil water availability, a process known as hydrotropism (Dietrich, 2018). Abscisic acid (ABA) and genes such as *MIZ1* and *MIZ2/GNOM* have been identified as key players in hydrotropic responses (Fromm, 2019). Overexpression of *MIZ1* has been shown to enhance root hydrotropism in *Arabidopsis* plants (Miyazawa et al., 2012). Therefore, its function is especially relevant under water-limiting conditions.

In this same context, ABA is a crucial phytohormone in plant responses to abiotic stress, particularly for acclimation to drought and salinity. A key gene in its biosynthesis is *NCED3*, which encodes an oxygenase essential for ABA production under water-deficit conditions. At the signaling level, *ABI1* and *ABI2* act as negative regulators, encoding type 2C protein phosphatases (PP2C) that inhibit the pathway in the absence of ABA. In contrast, *SnRK2* kinases are activated upon ABA accumulation, leading to the phosphorylation of transcription factors that induce stress-tolerance genes, including those encoding LEA proteins (protects cellular structures), antioxidants, and osmoprotectants. This hormonal pathway enables plants to adapt and survive under adverse environmental conditions (Movahedi et al., 2023).

Ethylene, often referred to as the “stress hormone,” is a gaseous regulator of plant growth that participates in multiple physiological processes. Its biosynthesis increases markedly in response to stress and during key developmental stages such as root elongation, leaf and flower senescence, seed germination, and cell differentiation. In rice and other species, ethylene signaling is tightly regulated at both physiological and molecular levels. Under adverse conditions, this pathway adjusts gene expression by upregulating or downregulating various stress-related gene families (Gamalero et al., 2023). Transcription factors, which are regulatory proteins, play a central role in coordinating biological processes under both normal and stress conditions. In plants such as *O. sativa* L., the APETALA2/ethylene response factor (AP2/ERF) transcription factor has been identified as a key component of stress responses. According to previous work AP2/ERF factors respond to multiple types of stress, including submergence, cold, salinity, drought, and heavy metal toxicity (Abiri et al., 2017). Interestingly, AP2/ERF transcription factors are also regulated in response to pathogen attacks and modulate the expression of pathogenesis-related (PR) genes. PR proteins are widely distributed and conserved across vascular plants, although their presence, abundance, and specific types can vary (Wang et al., 2022). Figure 2 provides a general overview of the impact of climate change–driven abiotic stress on plant growth, along with the phytohormonal, signaling, antioxidant, and osmoprotectant responses that help mitigate its effects.

**FIGURE 2:**
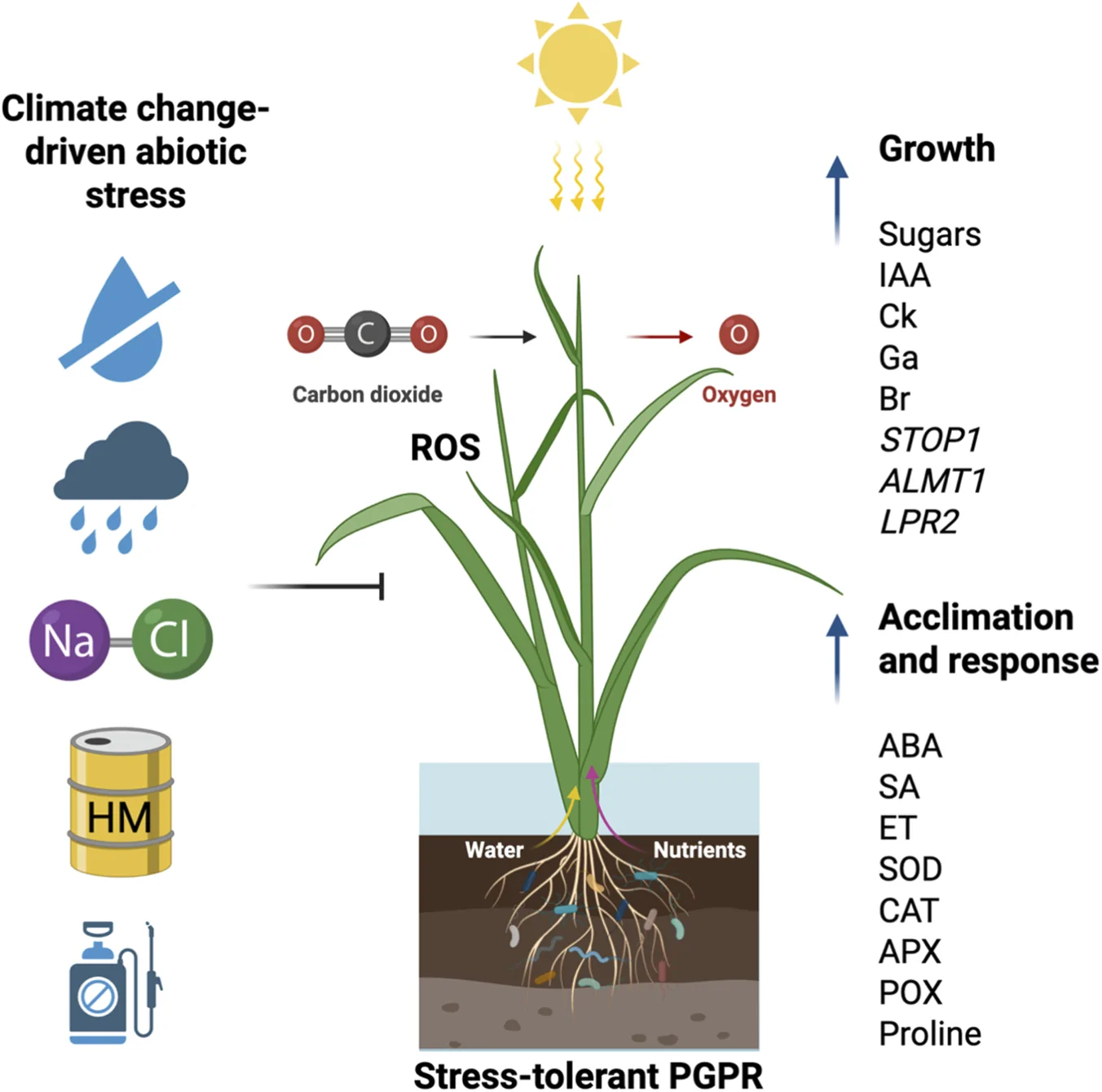
Abiotic and environmental stressors such as drought, flooding, salinity, heavy metals (HM), and pesticides negatively impact rice cultivation. Stress-tolerant PGPR promote growth, nutrient uptake, and adaptive responses. Indole-3-acetic acid (IAA); Cytokinins (CK); Gibberellic acid (GA); Brassinosteroids (BR); *STOP1* (a transcription factor); *ALMT1* (a malate transporter channel); *LPR2* (*LOW PHOSPHATE ROOT 2*); Abscisic acid (ABA), salicylic acid (SA), ethylene (ET), superoxide dismutase (SOD), catalase (CAT), ascorbate peroxidase (APX), and peroxidase (POX). See text for further details.

## 3 Bacterial stress tolerance and rhizosphere colonization under abiotic stress

Just as plants possess response mechanisms to cope with abiotic stress, PGPR also suffer from abiotic stress (Figure 1) and exhibit their own survival and adaptation strategies, including the production of osmoprotectant compounds and antioxidant enzymes (Valencia-Marin et al., 2024). Like plants, these bacteria respond by synthesizing compounds such as proline, glycine betaine, and trehalose. The latter is a non-reducing disaccharide in which two glucose units are linked by an α,α-(1,1)-glycosidic bond. In bacteria, the accumulation of this disaccharide has been widely associated with tolerance to various environmental stresses, including drought, cold, extreme heat, and salinity, where it may function as an osmoprotectant by contributing to the stabilization of cellular membranes (MacIntyre et al., 2022). Stronger genetic evidence has been reported in *Rhizobium etli*, a nitrogen-fixing symbiotic bacterium, in which mutants defective in trehalose production due to disruption of the *otsA* gene exhibited reduced tolerance to osmotic and desiccation stress. These findings support a role for trehalose biosynthesis in bacterial stress tolerance and suggest that this trait may influence the ability of *R. etli* to maintain effective symbiotic interactions with legumes such as common bean (*Phaseolus vulgaris* L.). (Suárez et al., 2008).

The production of reactive oxygen species (ROS) in bacteria is also a common response to environmental stress. However, ROS can cause severe damage, including the oxidation of iron-sulfur clusters in key metabolic enzymes, such as those involved in the Krebs cycle or the respiratory chain. Additionally, ROS lead to lipid peroxidation, protein damage, and DNA lesions (Seixas et al., 2022). In DNA, these lesions may include single- and double-strand breaks, thus making DNA repair systems such as homologous recombination and non-homologous end joining essential to prevent mutation accumulation and cell death. The synthesis of glutathione, ascorbic acid, and carotenoids, along with enzymes such as superoxide dismutase (SOD), catalase, and peroxiredoxins, plays a vital role in mitigating oxidative stress (Juan et al., 2021). Notably, bacteria that respond efficiently to oxidative stress tend to be better colonizers of competitive environments, such as the rhizosphere. Rhizosphere colonization is a critical step for PGPRs, as it enables them to deploy direct mechanisms, such as phytohormone production and enhanced nutrient uptake, and indirect mechanisms, such as pathogen biocontrol and induction of plant immune responses, to promote plant growth (Santoyo et al., 2021).

Other important bacterial strategies for survival and rhizosphere colonization include the production of exopolysaccharides and the modification of membrane lipids. In the first case, exopolysaccharides are part of the biofilm matrix, along with proteins, nucleic acids, and lipids (Barahona et al., 2010). In many cases, this biofilm also embeds microbial communities. Biofilm production has been extensively studied in PGPRs and is considered a key factor in root attachment-particularly in intimate root regions, such as the rhizoplane-and in the subsequent colonization of the endosphere. Some plant growth-promoting bacteria can colonize internal plant tissues and behave as endophytes. Endophytes are microorganisms that inhabit internal plant tissues for at least part of their life cycle without causing apparent disease symptoms; their interactions with the host can range from beneficial to neutral or context dependent (Bertani et al., 2016). Some endophytic bacteria can also be vertically transmitted through seeds to the next-generation (Walitang et al., 2017). In rice plants, multiple species of endophytic bacteria with beneficial activities-such as plant growth promotion and pathogen biocontrol-have been characterized (Bosco et al., 2024; Thampi et al., 2024; Wang et al., 2024). Likewise, stress-tolerant bacteria (*Priestia endophytica*, *Bacillus altitudinis*, and *P*. *megaterium*) have been isolated and characterized from the phyllosphere of rice plants, exhibiting functions that help their host mitigate abiotic stress (Arun et al., 2020).

Modification of membrane components (such as cardiolipin or phosphatidylethanolamine) is another survival strategy employed by bacteria in harsh environments. The membrane plays essential roles in maintaining fluidity, permeability, and structural integrity. However, under abiotic stress conditions such as desiccation, extreme temperature, or salt stress, the membrane is often among the first cellular components to be affected, leading to alterations in its structure, composition, and function, and ultimately disrupting cellular homeostasis (Willdigg and Helmann, 2021).

Recent studies suggest that some stress-tolerant *Bacillus* strains increase the production of phosphatidylethanolamine and branched fatty acids (16:1Δ9; 17:0i), as well as unsaturated fatty acids under saline stress conditions (Rojas-Solís et al., 2016). One phospholipid that has also been studied for its role in conferring bacterial tolerance and adaptation to various abiotic stresses, including salinity, is cardiolipin (CL). Mutants in the cardiolipin synthetase genes, *clsA* (Δ*clsA*) and *clsB* (Δ*clsB*), of *Pseudomonas fluorescens* UM270, which have reduced CL production, were negatively affected when exposed to saline conditions (100 and 200 mM NaCl). When these mutants were inoculated into tomato and maize plants, they failed to perform as effectively as the wild-type strain; they did not promote plant growth in parameters such as shoot length, chlorophyll content, and total plant dry weight (biomass). However, under non-saline (normal) conditions, the mutants were similarly effective as plant growth promoters compared to the wild-type strain (Rojas-Solis et al., 2023). In this context, cardiolipin (CL) production plays a key role when PGPRs are exposed to abiotic stresses such as salinity, since maintaining membrane fluidity and integrity is essential for the survival of bacteria (and plants) in soils affected by diverse abiotic stressors (Liu et al., 2019).

It is worth noting that species within the phylum *Bacillota* (e.g., *Bacillus* spp., *Clostridium* spp., *Sporosarcina* spp., and *Paenibacillus* spp.) can survive through sporulation; however, because this is not a metabolically active state, their plant growth-promoting functions and interactions with the plant are not active (Freire et al., 2025; Gohari et al., 2024). While dormancy is not considered a relevant state for active colonization or interaction with the plant host, it is a critical survival mechanism for withstanding abiotic stresses such as fire and extreme heat. Therefore, survival under stress should not be considered sufficient evidence of effective plant–microbe interactions or plant growth promotion. Before field application, PGPR strains should be pre-evaluated for their ability to actively colonize and persist in the rhizosphere under the target environmental conditions and to maintain their plant growth-promoting functions. In addition, strains showing consistent performance under controlled conditions should be further validated under field conditions, where environmental variability and complex soil and microbial interactions can strongly influence colonization, activity, and plant responses. Such pre-evaluation and field validation may improve the reliability and consistency of PGPR performance and facilitate the selection of strains with greater potential for effective agricultural application.

## 4 Application of stress-tolerant PGPR in rice systems

Rice cultivation requires specific conditions of waterlogged soils and, unfortunately, it is highly affected by climate variations, even more so than other cereal crops. When grown under rainfed conditions, both in upland and lowland areas, rice occupies around 45% of the total cultivated area and contributes approximately 25% to global production. Additionally, rice can suffer from water stress even when soil water content exceeds field capacity. Studies suggest that, due to future global warming, irrigated rice production could decrease by 7% by the year 2050. Meanwhile, rainfed rice yields are projected to decline by up to 6%, or more conservatively, around 2.5% (Saud et al., 2022). In research conducted by Zhang et al. (2020), it was observed that increases in temperature (approximately 1 °C–2 °C) caused a decrease in rice production in the field, with an impact of about 4–23 percent in 2015, and between 31 and 62 percent in 2016, depending on the type of crop and the heating method used.

One strategy to address the various impacts of abiotic stress on rice is to develop new varieties adapted to multiple farming systems and environmental conditions. For instance, China-one of the world’s leading producers-cultivates various types, including southern dual-cropping rice, Yangtze River basin rice, medium-cultivation rice, and (Zhang et al., 2020). However, in other parts of the world, these varieties are not well known, and if they could be adapted and cultivated under local systems, they might offer new opportunities.

Abiotic factors have severe consequences for rice crops, including growth inhibition, reduced photosynthetic capacity, poor germination, seedling mortality, and diminished biomass and grain quality (Sarma et al., 2023). To counteract these abiotic and biotic stressors, rice cultivation has traditionally relied on fertilizers, pesticides, and other agrochemicals-particularly during the Green Revolution, which increased yields unsustainably and contributed to long-term contamination of agricultural soils. Therefore, implementing innovative microbial strategies that minimize environmental and health impacts is urgent.

## 5 Drought stress

Rising temperatures lead to water scarcity in rice cultivation, severely affecting plant development and grain yield, given the crop’s high water demand and specific cultivation conditions. Rice is a water-intensive crop, and approximately 50% of its global cultivated area relies on irrigation systems. Some authors suggest that severe water stress in rice can lead to economic yield losses ranging from 48% to 94% during the reproductive stage and up to 60% during the grain-filling stage (Kim et al., 2020). Therefore, global rice production largely depends on the availability and accessibility of freshwater. Agriculture worldwide-not just rice cultivation-also heavily relies on water resources. In many regions, water scarcity poses a major constraint, making the development of drought-adapted crops an essential strategy to mitigate this stress.

Although rice is generally poorly adapted to drought, some genotypes have shown advantages by developing early root growth or by penetrating compact soil layers, improving water uptake. Proper root structure, density, and reach are key factors for accessing deep water, while root hairs and active root tips further contribute to absorption. In rice, the root system is the main organ responsible for water and nutrient uptake, making processes such as hydrotropism crucial for detecting soil moisture variations and essential for plant survival under drought conditions (Yamazaki et al., 2024). Modifying specific root traits can significantly enhance a plant’s ability to acquire water and nutrients, improving yields under stress. Traits such as increased root depth and thickness promote drought tolerance in some rice varieties, whereas others respond to drought by shortening their life cycle (Afonso et al., 2025).

A study by Ambreetha et al. (2018) explored the ability of a *Bacillus altitudinis* strain FD48 to mitigate drought effects in rice plants by modifying root architecture. The authors assessed multiple root parameters and found that PGPR inoculation significantly increased root number, lateral root number, root area, thickness, volume, and root bushiness. This strain also modulated IAA production in roots, confirmed by changes in the expression of auxin-responsive genes (AUX/IAA). Interestingly, genes involved in lateral root formation (OsIAA11 and OsIAA13) and primary root shortening (OsIAA1 and OsIAA4) were also modulated by *B. altitudinis* inoculation. This study is relevant because it enhances our understanding of how root systems interact with PGPRs to alleviate water-deficiency stress in rice plants.

Drought stress also affects other key physiological processes, particularly photosynthesis. A recent review highlighted that drought disrupts both light-dependent and carbon-fixation processes, promotes stomatal closure and oxidative stress, and triggers physiological and structural adjustments that help plants maintain photosynthetic activity under water deficit. Under drought conditions, plants undergo morphological changes, including reduced leaf area and number, accelerated senescence, and altered growth and development. Physiologically, drought impacts photosynthesis, transpiration, and osmotic balance. Moreover, genes involved in drought stress responses are strongly expressed in plants exposed to water deficit, while the associated microbiome can modulate the expression of these genes, potentially contributing to plant stress tolerance and adaptation (Qiao et al., 2024). For example, a recent study evaluated two osmotic stress-tolerant strains of *P. megaterium* (CACC109 and CACC119) in rice under drought stress (Lee et al., 2024). *In vitro* and pot experiments showed that both strains significantly improved seed germination of *O. sativa* L. under osmotic stress and stimulated root growth under both stressed and non-stressed conditions. In pot trials, the Bacilli strains also promoted overall plant growth, as evidenced by increased root and shoot length and weight, and broader leaves. Additionally, these strains increased chlorophyll content and proline synthesis in stressed plants compared to uninoculated controls. To confirm their effects on morpho-physiological parameters, drought-responsive genes, including *WRKY47*, *ZIP23*, *DREB2*, *NAC066*, *AREB1*, and *AREB2*, showed increased expression upon inoculation. Additionally, oxidative stress-related genes, such as *CAT*, *POD*, *APX*, and *SOD*, were also upregulated in *O. sativa* L. inoculated with stress-tolerant *Bacillus* strains.

Similar responses have been reported with other phyllosphere-associated *Bacillus* strains, such as *B. endophyticus* PB3, *B. altitudinis* PB46, and *P. megaterium* PB50. Notably, foliar application of strain PB50 significantly upregulated the expression of drought-related genes, including *LEA, RAB16B, HSP70, SNAC1,* and *bZIP23*, thereby improving rice tolerance to drought stress (Devarajan et al., 2021).

The modulation of morpho-physiological parameters, growth promotion, enhanced antioxidant responses, and the upregulation of drought-responsive genes following PGPR inoculation have also been observed in other economically important crops such as wheat (*Triticum aestivum* L.) (Ali and Maitra Pati, 2024), maize (*Zea mays* L.) (Kálmán et al., 2024), pepper (*Capsicum annuum* L.) (Lim and Kim, 2013), chickpea (*Cicer arietinum* L.) (Khan et al., 2019), Persian walnut (*Juglans regia* L.) (Lotfi et al., 2022), and pearl millet (*Pennisetum glaucum*) (Murali et al., 2021), and barley (*Hordeum vulgare* L.) (Alsamadany et al., 2024). Analysis of 57 studies evaluated the morphological, physiological, and biochemical responses of various plant species inoculated with PGPRs under drought conditions. The analysis concluded that PGPRs play a fundamental role in enhancing plant drought tolerance, supporting their use as bioinoculants to promote plant growth under water-limited conditions.

## 6 Salinity stress

The intrusion of seawater into coastal areas-driven by rising sea levels, freshwater scarcity, and the use of saline water for irrigation-induces osmotic and ionic stress in plants. Given the overlap in physiological and molecular responses between drought and other abiotic stresses, the application of multi-stress-tolerant PGPR represents a promising strategy to mitigate both types of stress (Alsamadany et al., 2024).

For example, in a recent study, 50 bacterial isolates were obtained from saline Pokkali rice fields in Kerala, South India. Among them, the *Pseudomonas taiwanensis* PK7 stood out for its tolerance to salinity stress. The effectiveness of strain PK7 as a PGPR was evaluated by inoculating it into both salt-sensitive and salt-tolerant rice varieties. The inoculation enhanced seedling growth, chlorophyll concentration, and proline content, indicating improved salt stress resistance in rice plants (Reshma and Dileep, 2025).

Another recent study demonstrated that the rhizobacterium *Bacillus amyloliquefaciens* SN13 modulated the expression of numerous genes involved in photosynthesis, hormone biosynthesis, and stress responses under salt stress. In addition, SN13 inoculation was associated with the upregulation of genes involved in lipid metabolism and cell wall biosynthesis (Chauhan et al., 2019). These findings suggest its potential role in mitigating the harmful effects of salinity.

When evaluating possible mechanisms involved in plant growth promotion and the induction of drought and salinity tolerance, it was observed that strain SN13 altered the metabolism of rice plants, particularly affecting fatty acids (dodecanoic, eicosenoic, hexadecanoic, linolenic, and octadecadienoic acids) and carbohydrates (glucose, galactose, fructose, ribose, trehalose, turanose, hexapyranose, and xylose) (Joshi et al., 2020). Notably, plants inoculated with this stress-tolerant strain performed better under both abiotic stresses.

Plants interact with their microbiome through three main zones: the rhizosphere, endosphere, and phyllosphere (Devarajan et al., 2021). Therefore, stress-tolerant strains with plant growth-promoting activity can potentially be isolated from any of these plant-associated regions. For instance, two salt-tolerant growth-promoting strains, *Pantoea stewartii* A and *Rossellomorea marisflavi* Y25, were isolated from the phyllosphere of mangrove plants growing in high-salinity environments of coastal intertidal zones. These saline ecosystems allow beneficial bacteria to become pre-adapted to salinity, evolving innate tolerance through natural selection. Both strains showed strong potential as rice bioinoculants for saline soils, as they demonstrated excellent ability to modulate osmotic substances in rice and upregulate the expression of salt-resistance-associated genes (Yang et al., 2024).

Several halotolerant PGPR species have shown a strong ability to promote rice plant growth. For instance, *Stutzerimonas stutzeri* and *Klebsiella pneumoniae* isolates promoted rice growth under salinity stress, confirming their capacity as halotolerant PGPR. Among other results, these three isolates exhibited high potential for indole-3-acetic acid (IAA) production and nitrogenase activity (Khumairah et al., 2022). ACC Deaminase in halotolerant PGPR during abiotic stress, including salinity, plants overproduce ethylene, leading to leaf yellowing, senescence of various organs, abscission of leaves, petals, and flowers, and, in severe cases, premature plant death (Gamalero et al., 2023). Ethylene biosynthesis in plants begins with the formation of S-adenosylmethionine (SAM), which is synthesized from methionine and ATP by the enzyme SAM synthetase, also known as AdoMet synthetase. SAM is then converted into 1-aminocyclopropane-1-carboxylic acid (ACC) by ACC synthase, and ACC is subsequently converted into ethylene by ACC oxidase. This final step also generates other volatile by-products, including hydrogen cyanide and carbon dioxide (Orozco-Mosqueda et al., 2020).

Stress-tolerant PGPR help mitigate this “plant stress” caused by excessive ethylene-responsible for growth inhibition-by degrading ACC via ACC deaminase (Figure 3). This enzyme breaks down ACC (the ethylene precursor), producing α-ketobutyrate and ammonia. ACC deaminase activity is one of the most studied traits in stress-tolerant PGPR, and for decades it has been associated with beneficial effects on plant growth under stress conditions, including salinity (Ali et al., 2014). Notably, ACC deaminase is not an uncommon feature limited to a few PGPR strains-it is commonly found in bacterial isolates and in other microbial groups, such as *Trichoderma* spp., which are mycoparasitic and promote plant growth (Timmusk et al., 2011).

**FIGURE 3:**
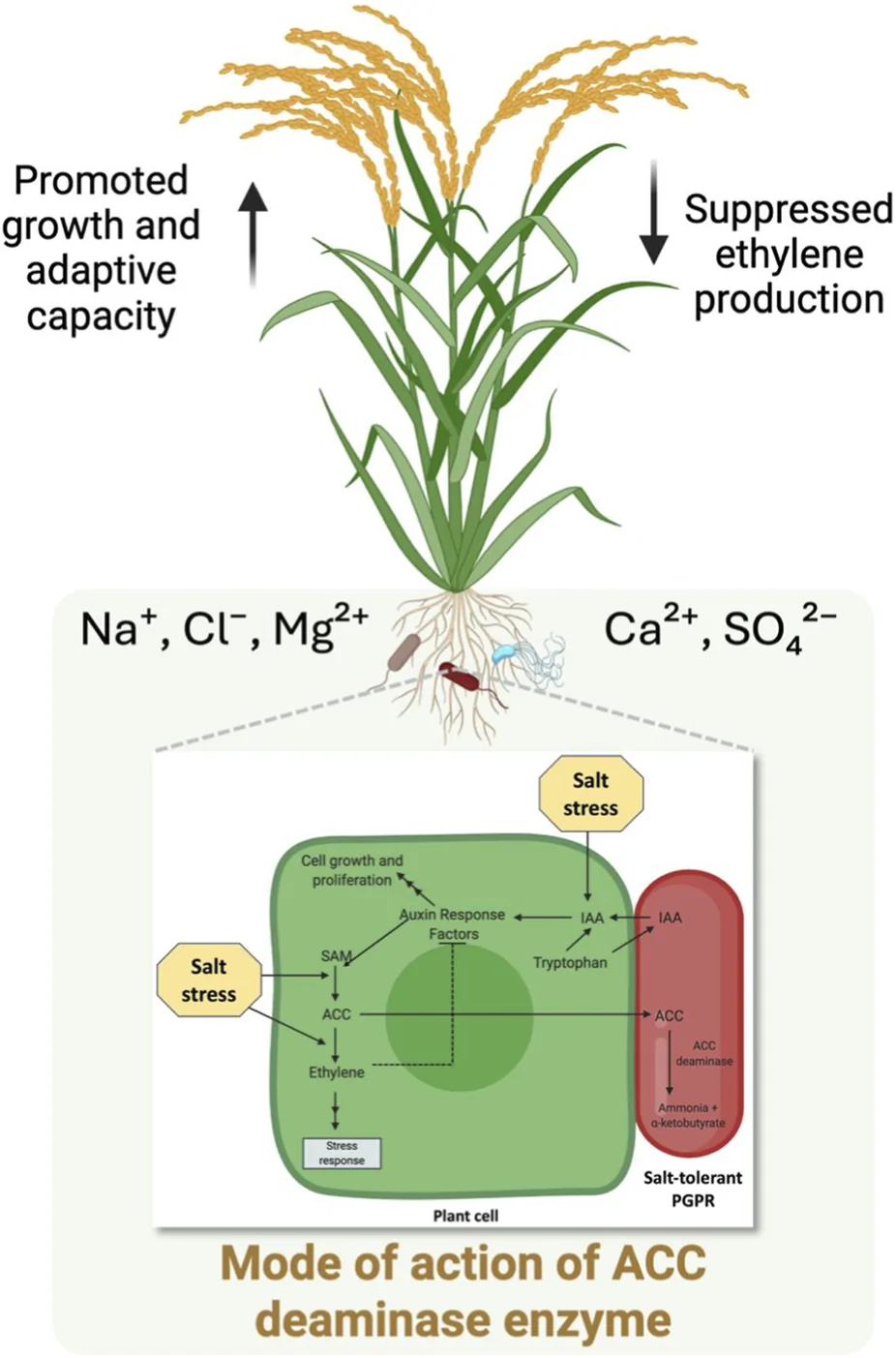
Mechanism of ACC deaminase-mediated reduction of ethylene levels in plants under saline stress and other abiotic stresses (e.g., drought, flooding, and heavy metal exposure) by stress-tolerant PGPR. Proposed mechanism by which stress-tolerant plant growth-promoting rhizobacteria (PGPR) enhance plant growth through ACC deaminase activity. In plants, S-adenosyl methionine (SAM) is converted into ACC by ACC synthase, and ACC is subsequently converted to ethylene by ACC oxidase. ACC deaminase-producing PGPR degrade ACC into α-ketobutyrate and ammonia, reducing ethylene biosynthesis and alleviating stress-induced growth inhibition. Modified from [17].

Evidence for the beneficial role of ACC deaminase under salt stress comes from studies on *acdS* gene mutants in two *Pseudomonas* strains, *P. fluorescens* YsS6 and *P. migulae* 8R6 (Ali et al., 2014). These mutants failed to promote tomato plant growth under salinity stress, in contrast to the wild-type strains with functional *acdS* genes, leading to the conclusion that ACC deaminase is directly responsible for the plant’s improved response to salinity.

In a more recent study, an ACCD gene (EF011160) retrieved from NCBI was synthesized and heterologously expressed in *Escherichia coli*, a non-PGPR enterobacterium. This engineered strain, named BL-EF, exhibited catalytic ACC deaminase activity. Interestingly, BL-EF showed plant growth-promoting traits under salt stress, significantly enhancing photosynthesis, increasing osmoregulatory compounds such as proline, and boosting antioxidant enzyme activities (SOD, CAT, and POD) in tomato plants. Additionally, BL-EF improved soil quality by solubilizing nutrients such as calcium and magnesium and facilitating their uptake by plants (Jia et al., 2025). This study highlights the importance of ACC deaminase in stress-tolerant PGPR, a key factor in enhancing plant tolerance and growth under abiotic stress. Further research should explore whether BL-EF is effective under rice-growing conditions and evaluate its rhizosphere colonisation potential, biofilm-forming ability, and other PGPR traits.

Rice cultivation can be adversely affected by salt stress, reducing growth and grain production. Strains possessing ACC deaminase activity have also demonstrated their ability to promote rice growth and alleviate salt stress (Ali et al., 2014). For instance, Bharti et al. (2024) isolated and applied a salt-tolerant endophytic bacterium identified as *Bacillus altitudinis* NKA32, isolated from rice roots. This strain exhibited multiple plant growth-promoting (PGP) traits, including IAA and siderophore production, zinc and phosphate solubilization, and ACC deaminase activity. In pot experiments with rice under salinity stress (300 and 600 mM NaCl), strain NKA32 improved several plant parameters, including fresh and dry weight, total chlorophyll content, total phenols and flavonoids, as well as protein and carbohydrate content, relative water content, and grain yield. Notably, ethylene levels in salt-stressed, inoculated plants were reduced by up to 71.53%, highlighting the role of ACC deaminase in mitigating stress and promoting growth.

Similar benefits of ACC deaminase activity in stress-tolerant PGPR with the capacity to alleviate salinity stress and promote plant growth—mainly in rice—have recently been reported for several species, including *Enterobacter asburiae* strain D2 (Ning et al., 2024), *Enterobacter* sp. JIV1 (Ji et al., 2024), *Enterobacter* sp. PR14 (Sagar et al., 2020), *Pseudomonas promysalinigenes* RL-WG26 (Ren et al., 2024), *Pseudomonas soyae* R600, *Bacillus haynesii* SFO145, *Salinicola halophilus* SFO075, and *Staphylococcus petrasii* SFO132 (Alonazi et al., 2025), *Pseudomonas atacamensis* KSS-6 (Arora et al., 2024), *Enterobacter aerogenes* P8, *Shinella* sp. R18, and *Mesorhizobium* sp. S1-7 (Sarin et al., 2025), *Ochrobactrum pseudogrignonense* P14 and *Arthrobacter woluwensis* M1R2 (Sobahan et al., 2025), *Serratia marcescens* VIRS2 (Ho et al., 2025), *Delftia tsuruhatensis* DYX29 and *Kandelia obovata* (Dai et al., 2025), *Pantoea agglomerans* FAP10 (Ansari et al., 2024), *Bacillus aryabhattai* strain PM34 (Azeem et al., 2024), *Alcaligenes faecalis* SSP8 (Vimal et al., 2024), *Halomonas arcis*, *Marinococcus tarigensis*, *Halobacillus dabanensis*, and *Halomonas eurihalina* (TG strains) (Atici et al., 2025), *Priestia* sp. HL3, *Bacillus* sp. HL6 and HG12, and *Paenibacillus* sp. HG24 (Ma et al., 2024), *Streptomyces* sp. GMKU 336 (Jaemsaeng et al., 2018), *Pseudomonas aeruginosa* GKP KS2_7 and *Bacillus subtilis* MBD 133 (Gupta and Pandey, 2019), *Bacillus subtilis* NBRI 28B, *B. subtilis* NBRI 33 N, and *B. safensis* NBRI 12 M (Misra and Chauhan, 2020), among others.

Importantly, stress-tolerant PGPR not only reduce ethylene levels via ACCD activity but also confer additional benefits that enhance plant growth under salinity stress. These include improvements in physiological processes, ion homeostasis, antioxidant defense systems, and the upregulation of growth-promoting hormones.

The search for new stress-tolerant PGPR strains as bioinoculants to improve plant stress responses and enhance growth continues. One promising strategy is the isolation of PGPR adapted to saline conditions. Such is the case of the newly proposed species “*Aquabacter pokkalii”* L1I39, isolated from saline habitats in India (specifically from coastal agricultural fields affected by salinity). This strain is notable for exhibiting positive ACC deaminase activity and nitrogen fixation, and for promoting Pokkali rice growth under nitrogen-deficient seawater conditions (Sunithakumari et al., 2024). This work underscores the untapped microbial diversity of stress-tolerant PGPR that may serve as effective bioinoculants in rice-growing regions affected by salinity.

## 7 Flooding and submergence stress

Although rice is cultivated in flooded soils, unexpected or prolonged submergence can severely damage the crop. When water levels rise above seedling height or remain high for several days, oxygen availability in the soil drops, limiting root respiration and causing physiological stress. The low oxygen levels disrupt root energy metabolism by inhibiting the tricarboxylic acid (TCA) cycle in mitochondria while activating glycolysis and fermentation, leading to the accumulation of ethanol—a highly toxic compound (Yu et al., 2021). Additionally, the buildup of abscisic acid (ABA) in the leaves promotes stomatal closure, resulting in reduced CO_2_ uptake and carbohydrate production due to impaired photosynthesis. Stomatal closure can also be triggered by the accumulation of ethylene in aerial tissues, which is rapidly synthesized under hypoxic conditions in roots or derived from its precursor ACC in the leaves (Hsu et al., 2021). Other negative effects of ethylene include the suppression of leaf growth and the development of leaf epinasty. Furthermore, flooding can leach essential nutrients, promote disease and pest outbreaks, and hinder seed germination and early plant development. These conditions, especially during critical stages such as flowering, can significantly reduce yield (Aslam et al., 2023).

According to Zölch et al. (2018) there are two main strategies to manage flood stress in rice cultivation: traditional water management infrastructure—such as drainage systems and dikes—and nature-based solutions like using landscape features and living organisms, including stress-tolerant PGPR. These beneficial bacteria, particularly those adapted to flooded environments, may help induce tolerance mechanisms and promote adaptive physiological responses in plants.

In a study by Bal and Adhya (2021), four PGPR strains with ACC deaminase activity (*Bacillus* sp. AR-ACC1, *Microbacterium* sp. AR-ACC2, *Methylophaga* sp. AR-ACC, and *Paenibacillus* sp. ANR-ACC3) were inoculated into rice seeds subjected to submergence stress, resulting in increased germination rates. Other growth parameters also improved with PGPR application, including seedling vigor index (SVI), root and shoot length, and total chlorophyll content, compared to non-inoculated seeds submerged under the same conditions. One notable isolate, *Microbacterium* sp. AR-ACC2, reduced ethylene levels by approximately 85% in submerged rice seedlings compared to untreated controls. This finding is especially relevant considering that, in India, over five million hectares of rainfed lowland rice fields are affected by flooding, causing significant losses in rice productivity (Sandhu et al., 2019).The use of ACC deaminase-producing bacteria thus presents a promising strategy for mitigating flood-related stress.

Among the most abundant plant growth-promoting genera in soils is *Pseudomonas* (Guzmán-Guzmán and Santoyo, 2022). Several *Pseudomonas* species possess stress tolerance traits, including the ability to survive in flooded rice soils. In a recent study, the benefits of three rhizospheric *Pseudomonas* isolates (two *P. putida* and one *P. chorii*) were evaluated in rice plants grown in flooded soils. Under these environmental conditions, the pseudomonads promoted root and shoot growth. These isolates exhibited multiple mechanisms, including the production of siderophores, proteases, cellulases, ammonium, tricalcium phosphate solubilization, nitrogen fixation, synthesis of IAA, salicylic acid, and hydrogen cyanide (HCN). Given that some of these traits are also associated with pathogen suppression, the study found that plants inoculated with these stress-tolerant PGPRs inhibited the growth of several fungal pathogens such as *Xanthomonas oryzae*, *Magnaporthe grisea, Aspergillus niger, Fusarium oxysporum, Helminthosporium* sp., *Curvularia* sp., and *Rhizoctonia solani* (Das et al., 2022). The authors concluded that these stress-tolerant PGPR species not only support rice growth but also help mitigate the impact of various biotic stressors.

## 8 Mitigation of methane emissions in rice systems

Methane (CH_4_) emissions from rice cultivation are a significant concern due to their contribution as a potent greenhouse gas driving climate change, prompting the development and implementation of various strategies to reduce their release (Zhang et al., 2016). For instance, soil amendments, organic matter management, crop rotation, cultivar selection, different tillage methods, and water management practices have been proposed as key approaches in recent years. In particular, promoting midseason soil aeration through short-term drainage has emerged as one of the most effective tactics (Yagi et al., 1997). However, these measures alone have proven insufficient, and microbial applications are now needed to reduce CH_4_ emissions in paddy soils further.

Stress-tolerant PGPRs not only benefit their rice hosts but also help reduce methane emissions in paddy soils, where methanogenic bacteria thrive under anaerobic conditions and produce this greenhouse gas. A notable example is *Paenibacillus yonginensis* DCY84T, which, when coated onto rice seeds, significantly reduces methane emissions in cultivated fields. One proposed mechanism is that DCY84T modulates the expression of the *mxaF* gene, which encodes methanol dehydrogenase, an enzyme that oxidizes methanol produced during the initial stage of methane degradation. Notably, the addition of silicate to DCY84T-coated rice seeds enhanced the abundance of bacterial families such as *Anaerolinaceae*, Clostridiaceae, and *Nitrospirae*, which compete with methanogens for organic substrates, thereby reducing methane production (Justine et al., 2025). Altogether, *P. yonginensis* DCY84T may help reduce greenhouse gas emissions associated with rice cultivation and, potentially in combination with other stress-tolerant PGPR, offer dual benefits for plant health and environmental sustainability in rice farming systems.

PGPR capable of utilizing methane in flooded paddy ecosystems represent a promising strategy to mitigate greenhouse-gas emissions while simultaneously benefiting rice crops through their plant growth-promoting (PGP) mechanisms. Under flooded conditions, anaerobic decomposition promotes methanogenesis, resulting in CH_4_ production that can diffuse toward the rice rhizosphere and reach the atmosphere through root aerenchyma. Methane-utilizing bacteria can intercept and oxidize CH_4_, primarily through methane monooxygenase activity, thereby potentially reducing CH_4_ emissions. At the same time, these bacteria may promote plant growth through mechanisms such as nutrient mobilization, phytohormone modulation, and regulation of plant stress responses. The combination of methane oxidation and PGP activities highlights their potential multifunctionality in rice agroecosystems. However, their effectiveness depends on their ability to colonize the rhizosphere and maintain both methane-oxidizing and PGP activities under flooded field conditions (Justine et al., 2025; Rani et al., 2021). This potential was demonstrated in the study by (Rani et al., 2021), who isolated and applied methane-utilising bacteria (MUB) with PGP traits, naturally adapted to flooded rice fields. The authors obtained 30 isolates from five rice-growing regions in India, including *Hyphomicrobium*, *Burkholderia*, *Methylobacterium*, *Paenibacillus*, *Pseudomonas*, *Rahnella*, *Meyerozyma*, and *Pyricularia oryzae* (syn. *Magnaporthe oryzae*). These isolates showed significantly greater methane utilisation than other microbial strains. In addition to their potential to reduce methane emissions in rice soils, several isolates also exhibited beneficial traits for plant development, such as solubilization of phosphorus, potassium, and zinc, nitrogen fixation, and the synthesis of the auxin indole-3-acetic acid (IAA).

The regions studied by the authors are particularly interesting due to their diverse edaphic and climatic characteristics. For instance, in the Lower Brahmaputra Valley in Assam, *Sali* rainfed rice is cultivated in lowland areas prone to flooding. In Kochi, Ernakulam District, flooded lowlands are used for integrated rice-fish farming systems. In Tamil Nadu, at the regional station of the Indian Agricultural Research Institute in Aduthurai, rice–rice monoculture is practiced in medium lands under both rainfed and irrigated conditions. In the Indo-Gangetic Plain of Varanasi, Uttar Pradesh, an upland irrigated rice–wheat rotation system is used. Lastly, in Gaya, Bihar-also part of the Indo-Gangetic Plain-rice–wheat cropping is carried out in drought-prone upland irrigated fields (Rani et al., 2021).

It is worth noting that, although a wide range of environmental, soil, and climatic conditions likely exists in other parts of the world, their potential for rice cultivation remains largely unexplored. This not only limits the expansion of rice production but also hinders research on the application of stress-tolerant PGPR in productive agroecosystems. Therefore, promoting rice cultivation in similarly diverse regions of Latin America, alongside expanded research and implementation of these beneficial microbial strategies, would be advisable.

## 9 Heavy metals and synthetic pesticides

Exposure of plants to heavy metals (HMs) and trace metals causes multiple forms of physiological and biochemical damage, including inhibition of root growth, chlorosis, impaired photosynthesis, and excessive generation of reactive oxygen species (ROS). These toxic HMs interfere with the uptake of essential nutrients and damage key cellular structures, compromising plant development and reducing grain yield in rice (Nowicka, 2022).

Rice cultivation is particularly susceptible to contamination by heavy metals such as cadmium (Cd), chromium (Cr), lead (Pb), and mercury (Hg), which can accumulate in plant tissues, including grains. Other metalloids, such as arsenic (As), are also major contaminants, especially in countries like India, which face significant challenges in reducing the levels of heavy metals in groundwater and soils used for rice cultivation to non-toxic levels (Majhi et al., 2025). These HMs, derived from natural or anthropogenic sources such as chemical fertilisers, wastewater, and mining activities, negatively affect rice growth, photosynthesis, and nutrient uptake. In addition to lowering crop yield, they pose a significant health risk by entering the food chain. Moreover, HMs alter the rhizosphere microbiota and its ability to interact beneficially with the plant, selecting for HM-resistant microorganisms and competing with PGPRs (Gupta et al., 2023).

Fortunately, certain contaminated agroecosystems such as those affected by As can act as valuable sources for isolating As-tolerant PGPR. This is demonstrated by the recent study by Majhi and colleagues (2025), who isolated a total of 663 bacteria from rice field soils with varying As levels across five districts of West Bengal, India. A preliminary selection of 10 isolates showed good tolerance to arsenite (As-III) and arsenate (As-V), as well as multiple PGP traits (biofilm formation, indole-3-acetic acid production, siderophore production, and phosphate solubilization). Two of these isolates, *Priestia flexa* NBRI4As1 and *Pseudomonas chengduensis* NBRI12As1, not only tolerated arsenite and arsenate, but also promoted rice plant growth and alleviated arsenic stress under greenhouse conditions.

In some cases, rice-growing soils may not only be contaminated with HMs, but additional stressors may synergize to challenge plant growth and productivity. For instance, two endophytic isolates resistant to both metals and salt stress, *Brucella pseudogrignonensis* P14 and *A. woluwensis* M1R2, were shown to stimulate rice growth under saline and Cr-stressed conditions. The application of these stress-tolerant PGPR significantly reduced the uptake of Na^+^ and Cr^6+^ in rice, with *A. woluwensis* M1R2 showing remarkable efficiency by decreasing Cr^6+^ translocation by 38% and its bioaccumulation by 42% under combined stress (NaCl + Cr) (Sobahan et al., 2025).

Another example of combined stress involving pesticide exposure, salinity, and drought illustrates the potential of stress tolerant PGPR. Four pesticide tolerant isolates, *Enterobacter cancerogenus* E50S2 4, *Bacillus amyloliquefaciens* E50S2 3, *Klebsiella quasipneumoniae* M50R2 3, and *Bacillus velezensis* M100S1 4, exhibited strong capacity to degrade 2,4-D and mancozeb residues in soil. These results are particularly relevant, as the isolates could be used for bioremediation of pesticide contaminated soils while simultaneously alleviating abiotic stresses such as salinity and drought (Ma et al., 2023).

PGPR play an important role in remediating heavy metal–contaminated environments through diverse mechanisms, including efflux systems, siderophore production (mainly iron-chelating agents), other chelating compounds, biotransformation, biosorption, bioaccumulation, precipitation, biodegradation, and biomineralization. These bacteria have proven effective in bioremediating HM-contaminated soils, and ACC deaminase activity is also crucial for maintaining ethylene levels in plants at tolerable levels (Gupta et al., 2023).

For instance, one study evaluated the inoculation of three HM-tolerant and ACC deaminase–positive isolates in rice plants (variety Satabdi) grown under heavy metal stress (Pandey et al., 2013). The results showed increased germination rates and relative root elongation (RRE) compared to uninoculated plants. The toxic effects of metals were mitigated in the presence of these bacteria, and both total biomass and root/shoot ratio were enhanced with inoculation of these metal-tolerant PGPR.

In another study, inoculation with the ACC deaminase–producing strain *Klebsiella aerogenes* MCC 3092 showed beneficial effects on rice seedlings exposed to Cd, alleviating cytotoxic effects. This strain also exhibited PGP mechanisms such as nitrogen fixation, IAA production, and phosphate solubilization, which likely contributed to plant growth promotion, while its ACC deaminase activity may have helped reduce ethylene levels, alleviating Cd-induced stress (Pramanik et al., 2018).

The presence of multiple PGP mechanisms in stress-tolerant PGPR gives these bacteria a clear advantage in maintaining their beneficial effects on plants and surviving in HM-contaminated conditions. This was demonstrated by (Mendoza-Hernández et al., 2016), who evaluated four bacterial strains from the genera *Escherichia* (N16)*, Enterobacter* (K131), *Enterobacter* (N9), and *Serratia* (K120). These strains exhibited the ability to produce indole-3-acetic acid, siderophores, solubilize inorganic phosphate, and produce ACC deaminase. Among them, *Serratia* K120 was able to promote the growth of *Helianthus annuus* L. When exposed to HMs such as Pb, As, and Cu, the bacterial strains showed increased ACCD and IAA production, with *Serratia* K120 performing even better when grown in the presence of all analysed HMs (Cu, As, Pb, Ni, Cd, and Mn). These findings highlight *Serratia* K120 as a promising candidate for bioremediation and plant growth promotion, especially in rice fields affected by multi-metal contamination. Table 1 shows some works evidencing the role of stress-tolerant PGPR to ameliorate abiotic stress in rice plants.

**TABLE 1:** Recent studies on stress-tolerant PGPR benefiting rice growth and mitigating abiotic stress.

Stress-tolerant PGPR species	Strain(s)	Abiotic stress	Benefit provided to the plant	PGP mechanisms	References
*Bacillus altitudinis*	FD48	Drought	Increased the number of roots, lateral roots, root area, thickness, volume, and bushiness	Modulation of expression of auxin-responsive genes (AUX/IAA), genes involved in lateral root formation (OsIAA11 and OsIAA13) and primary root shortening (OsIAA1 and OsIAA4)	(Ambreetha et al., 2018)
*Priestia megaterium* (*Bacillus megaterium*)	CACC109 and CACC119	Drought	Both strains significantly improved seed germination, osmotic stress response and stimulated root growth	Phosphate solubilization, nitrogen fixation, indole-3-acetic acid (IAA) production, siderophore secretion, 1-aminocyclopropane-1-carboxylate deaminase activity, and exopolysaccharide production	(Lee et al., 2024)
*B. endophyticus, B. altitudinis, and B. megaterium* (Phyllosphere bacteria)	PB3, PB46 AND PB50	Drought	Upregulated the expression of drought-related genes (*LEA, RAB16B, HSP70, SNAC1*, and *bZIP23*)	Not determined	Devarajan et al., 2021
*Bacillus licheniformis, Bacillus haynesii, Bacillus paralicheniformis, Bacillus licheniformis, Bacillus paralicheniformis,* and *Bacillus haynesii*	A21, EN43, EN107, EN108, EN121, EN124	Drought	Higher biomass accumulation, increased grain yield and reversal of stress indicators	Differential activity bewteen the siolates to produce IAA, sideorphores, solubilizaci{on de P y Zn, ACC desaminase	(Joshi et al., 2020)
*Bacillus amyloliquefaciens*	SN13	Drought and salinity	Improved growth parameters, nutrient content, physio-biochemical traits and reduced the oxidative stress	ACC deaminase activity, production of biofilm, IAA, and phosphate solubilization	(Bisht et al., 2025)
*Pseudomonas atacamensis*	KSS-6	Salinity	Improved the nutrient content (P, K, Zn, Fe, Mg, and Mn) by more than 35% and enhanced proline, flavonoids, carbohydrates, protein, dietary fiber, and antioxidant content of rice grains. Plant growth promotion	Multiple plant growth-promoting traits and synergistic activity with organic manure	(Arora et al., 2024)
*Pseudomonas stutzeri* and *Klebsiella pneumoniae*	S3, S5	Salinity	Higher shoot height, root length, and plant dry weight	Production of IAA production and nitrogenase activity	(Khumairah et al., 2022)
*Pseudomonas taiwanensis*	PK7	Salinity	Enhanced seedling growth, chlorophyll concentration, and proline content	ACC deaminase activity and EPS production	(Reshma and Dileep, 2025)
*Bacillus altitudinis*	NKA32	Salinity	Improved fresh and dry weight, total chlorophyll, phenol and flavonoid contents; enhanced protein, carbohydrate, relative water content, and grain yield. Ethylene levels were reduced	Production of IAA and siderophores, zinc and phosphate solubilization, ACC deaminase activity	(Bharti et al., 2024)
*Ochrobactrum pseudogrignonense* and endophyte *Arthrobacter woluwensis*	P14, M1R2	Salinity and chromium	Alleviation of oxidative stress indicators—including reduced MDA, H_2_O_2_ content, and EL, along with increased osmolyte synthesis and antioxidant enzyme activity—was observed under single and dual stress conditions. Na^+^ and Cr^6+^ uptake was restricted	Positive for IAA, ACC-deaminase, P-solubilization	(Sobahan et al., 2025)
*Bacillus* sp., *Microbacterium* sp., *Methylophaga* sp., *Paenibacillus* sp	AR-ACC1, AR-ACC2, AR-ACC, ANR-ACC3	Submergence	Improved seedling vigor index (SVI), root and shoot length, and total chlorophyll content. Reduction in ethylene levels	ACC deaminase activity	(Bal and Adhya, 2021)
*Pseudomonas fluorescens*	REN1	Flooding	Reduced ethylene levels, endophytic colonization and root elongation	ACC deaminase, production of IAA, siderophores and siderophore production and phosphate solubilization	(Etesami et al., 2014)
*P. putida*, *P. chorii*	TB326, 325	Flooding	Plant growth promotion (root and shoot) and biocontrol of diverse phytopathogens	Solubilization of tricalcium phosphate, nitrogen fixation, and production of IAA, salicylic acid, hydrogen cyanide, siderophores, protease, cellulase, and ammonia	(Das et al., 2022)
*Priestia flexa* and *Pseudomonas chengduensis*	NBRI4As1, NBRI12As1	Arsenite and arsenate	Plant growth promotion and increased As tolerance	Biofilm formation, production of IAA, siderophores, and phosphate solubilization	(Majhi et al., 2025)
*Enterobacter cancerogenus*, *Bacillus amyloliquefaciens*, *Klebsiella quasipneumoniae*, and *Bacillus velezensis*	E50S2-4, E50S2-3, M50R2-3, M100S1-4	Pesticides, salinity, and drought	Degradation of 2,4-D and mancozeb. Mitigation of drought and salt stress	ACC deaminase activity, biofilm formation, and exopolysaccharide production	(Ma et al., 2023)
*Enterobacter aerogenes*	MCC 3092	Cadmium	Alleviation of cytotoxic effects and reduced ethylene levels	ACC deaminase, nitrogen fixation, IAA production, and phosphate solubilization	(Pramanik et al., 2018)
*Ochrobactrum* sp., *Bacillus* spp.	CdSP9, PbSP6, AsSP9	Cadmium, Arsenic, Lead	Germination percentage, relative root elongation (RRE), amylase and protease activities were increased. Metal toxicity amelioration	ACC deaminase	(Pandey et al., 2013)

## 10 Bioformulation and delivery of stress-tolerant PGPR

PGPR is primarily used in liquid form, offering an effective and cost-efficient approach to producing microbial inoculants. This method involves diluting the inoculant in water before applying it to seeds, planting furrows, or directly into soils (Benítez et al., 2025b). Despite their potential, the application of Gram-negative PGPR in agriculture remains limited. Liquid formulations, commonly used for field inoculation, often lead to decreased microbial viability due to desiccation and temperature fluctuations during storage and transport Gram-negative genus (*Azospirillum*, *Pseudomonas*, *Enterobacter*, *Serratia* sp.) are particularly sensitive to these conditions, which has restricted their commercial development compared to more robust Gram-positive bacteria (*Bacillus* sp.) (Benítez et al., 2024). Furthermore, liquid inoculants do not ensure PGPR viability once introduced into the soil, which is crucial for effective colonisation of the plant rhizosphere. As an alternative to liquid formulations, solid inoculants (Giraldo et al., 2023).

Bioencapsulation is designed to protect microorganisms introduced into the soil and to enable their gradual and prolonged release; even for stress-tolerant PGPR, this type of protection would provide an additional advantage for surviving in somewhat extreme edaphic environments (de Freitas et al., 2024). The rate of degradation of the encapsulation matrix is intrinsically linked to the biological activity of the microorganisms; a more rapid degradation of the matrix results in reduced protection and biological activity (Martin-Díaz et al., 2025). These dried beads or capsules can be stored at ambient temperature for extended periods, providing a favourable environment for bacteria and minimising the risk of reduced survival (de Bashan et al., 2024). By incorporating essential nutrients for PGPR growth, these inoculants can be improved, effectively transforming the capsules into bioreactors that release more encapsulated PGPR into the soil (Abd El-Mageed et al., 2022). The bioencapsulation of PGPR offers several advantages, including controlled release of PGPR into the soil, protection of microorganisms from biotic and abiotic stresses, and reduced contamination during storage and transport (Benítez et al., 2025a).

For effective bioencapsulation, the selected materials should exhibit key traits, including ease of handling during formulation, compatibility with PGPR, protection of PGPR from environmental stresses, controlled release of PGPR, support for biopolymer degradation, facilitation of the inoculation process, and maintenance of high cell viability in the formulations (Abd El-Mageed et al., 2022). The most common materials that meet these requirements are biopolymers, such as sodium alginate, carrageenan, Arabic gum, xanthan gum, starch, cellulose, and chitosan. Salts of sodium alginate and chitosan are the most commonly used because they are cheaper (Giraldo et al., 2023).

Various techniques are employed to encapsulate PGPR, including physical methods such as spray-drying, spray chilling/cooling, extrusion, and fluidised-bed processes. Chemical approaches such as co-crystallisation, molecular inclusion, or interfacial polymerisation are also used, along with physicochemical methods such as coacervation and gelation/inverse gelation. Bioencapsulation of PGPR is carried out through a three-step process. Initially, an active component is integrated into either a liquid or solid matrix. The next phase involves a mechanical procedure in which a solution is dispersed or sprayed onto solid particles while they are mechanically stirred. The final step consists in stabilising the droplet or pellet formed in the second stage by chemical polymerisation, physical-chemical methods such as gelation and coacervation, or physical processes such as evaporation and solidification (Benítez et al., 2024). Overall, the effectiveness of stress-tolerant PGPR depends not only on their ability to withstand abiotic stress but also on their survival, release, and active colonization of the rhizosphere after application. Therefore, bioformulations should be evaluated not only for bacterial viability and shelf life, but also for their ability to preserve PGP functions and promote consistent plant responses under field conditions. Integrating stress tolerance, formulation, rhizosphere colonization, and field validation is essential for developing reliable PGPR-based inoculants.

## 11 Challenges in the application of stress-tolerant PGPR

At present, the commercial success of bioencapsulation is constrained by significant limitations. Several obstacles hinder its widespread adoption, including the need to reduce production costs, improve scalability, and gain greater acceptance among producers. Addressing these challenges requires technological progress, industry collaboration, and extensive field research. Lowering costs can be achieved by optimising production processes using efficient raw materials. Automation and standardisation help reduce labour costs and enhance consistency, while modular production units should be designed to support various microorganisms (Schoebitz et al., 2013). Partnering with the traditional bio-input industry can accelerate scaling. Improvements in multi-injection and jet cutter techniques can enhance the efficiency and cost-effectiveness of the bioencapsulation process (Martin-Díaz et al., 2025). The development and application of stress-tolerant PGPR–based bioinoculants should consider both biosafety and economic feasibility. Rigorous biosafety assessments are also required to ensure environmental and human safety, while cost-effective production, formulation stability, scalability, and regulatory compliance are essential for successful commercialization (Khatoon et al., 2024).

Despite the proven potential of stress tolerant PGPR to enhance crop performance under adverse environmental conditions, several challenges limit their consistent and large scale application in rice based agroecosystems. One of the main constraints is the variable and often limited reproducibility of PGPR effects under field conditions. While many strains show strong and consistent benefits in predictive, laboratory and greenhouse experiments, their efficacy frequently decreases in open field environments, where rice is predominantly cultivated, due to complex interactions with native soil microbiota, highly dynamic abiotic factors, soil heterogeneity, and differences in crop management practices. As a result, the positive outcomes observed under controlled conditions are not always reproducible at the farm scale (Bal and Adhya., 2021; Zhang et al., 2016).

Farmer adoption and socioeconomic factors play a decisive role in the success of PGPR based technologies, particularly in rice farming systems where production is largely carried out under open field conditions and often by smallholder farmers. Limited awareness, lack of technical training, uncertainty about economic returns, and resistance to changing conventional practices can slow the uptake of microbial inoculants. Demonstration trials, extension services, and participatory research involving farmers are critical to build confidence and show the tangible benefits of PGPR under real rice growing conditions (Schoebitz et al., 2013). Integrating PGPR into broader sustainable management systems such as reduced chemical inputs, organic amendments, and diversified cropping will be key to maximizing their impact and ensuring long term adoption (Alonazi et al., 2025; Giraldo et al., 2023).

## 12 Conclusions and future perspectives

PGPR represent an efficient, cost-effective, and environmentally friendly alternative. Some of these bacteria also have the capacity to tolerate abiotic stress conditions—exacerbated by climate change—and support rice plants under such stresses. These stress-tolerant PGPR are therefore an excellent option for mitigating stresses such as flooding, salinity, drought, and heavy metal contamination, among others, which negatively impact rice production due to the crop’s sensitivity to environmental changes. Importantly, stress-tolerant PGPR may exhibit multifunctional traits because different abiotic stresses can induce partially overlapping physiological and molecular responses in both plants and bacteria (Khatoon et al., 2024). For example, drought and salinity can cause osmotic imbalance, oxidative stress, and alterations in cellular homeostasis, triggering common or complementary protective mechanisms. Consequently, PGPR capable of tolerating one type of stress may possess mechanisms that also contribute to plant protection under other stress conditions, although the effectiveness of these responses may depend on the specific stress and environmental context (Ansari et al., 2024). This multifunctionality could increase the potential of stress-tolerant PGPR for applications in agricultural systems exposed to multiple or simultaneous stresses. This is especially relevant today, as climate patterns have become increasingly unpredictable in regions highly vulnerable to climate change, leading to more frequent and intense episodes of drought, flooding, and salinity stress that severely threaten rice-based agroecosystems (Chauhan et al., 2023).

A major concern in rice cultivation (and in intercropping systems) is the combined effect of multiple stressors, where the application of stress-tolerant PGPR alone may not be sufficient to counteract the negative impacts on plants. Additional sustainable strategies will be required to restore the soil ecosystem, including organic amendments, reduced reliance on chemical inputs, crop rotation, intercropping, and the integration of microbial consortia to rebuild soil structure and function. As discussed in several studies, stresses such as drought combined with pesticide contamination have additive effects on plant growth. Pollution from other sources, such as air, also has detrimental effects-not only on crops but also on agricultural workers-calling for broader attention from both society and the scientific community (Chauhan et al., 2023; Sobahan et al., 2025).

Mutualistic and beneficial plant–bacteria relationships have existed for millions of years, so continuing the search for new stress-tolerant PGPR species with yet-unknown mechanisms is a pressing need. Likewise, exploring genetic modification techniques such as CRISPR-Cas9 could be a promising approach to enhance the expression of key genes, such as *acdS*, which encodes ACC deaminase and is present in several stress-tolerant PGPR species (Ali et al., 2014; Arun et al., 2020; Bharti et al., 2024). Of course, research on CRISPR-modified PGPR should adhere to strict biosafety and regulatory frameworks in every country where their application is intended. Risk assessments addressing off-target effects, horizontal gene transfer, and potential ecological impacts are required prior to open-field release or commercialization. Controlled experimentation, transparent reporting, and compliance with national and international regulations are essential to ensure responsible application. Finally, omics techniques-such as genomics, transcriptomics, and metabolomics-applied to study the impact of stress-tolerant PGPR on the native soil microbiome (and rhizomicrobiome) in rice cultivation areas could reveal new networks of interactions that we do not yet fully understand, but that may be crucial for sustainably stimulating plant resilience to abiotic stress from multiple perspectives.
